# Characterization of a Novel Food Grade Emulsion Stabilized by the By- Product Proteins Extracted From the Head of Giant Freshwater Prawn (*Macrobrachium rosenbergii*)

**DOI:** 10.3389/fnut.2021.676500

**Published:** 2021-06-25

**Authors:** Yi Wu, Yufeng Li, Ronghan Wang, Yong Zhao, Haiquan Liu, Jing Jing Wang

**Affiliations:** ^1^College of Food Science and Technology, Shanghai Ocean University, Shanghai, China; ^2^Laboratory of Quality and Safety Risk Assessment for Aquatic Products on Storage and Preservation (Shanghai), Ministry of Agriculture, Shanghai, China; ^3^Shanghai Engineering Research Center of Aquatic-Product Processing and Preservation, Shanghai, China; ^4^Department of Food Science, Foshan University, Foshan, China

**Keywords:** giant freshwater prawn, by-product, protein-stabilized emulsions, aggregation behavior, long-term stability, freeze-thaw stability

## Abstract

The aim of this work was to develop a food-grade emulsion that stabilized by the by-product proteins in the head of giant freshwater prawn. The physicochemical properties of protein particles were characterized, and the stability of proteins-stabilized emulsions under different environmental stresses was evaluated. Results showed that the proteins were relatively hydrophilic and preferentially resided in the aqueous phase to form oil/water emulsions. On this basis, the proteins exhibited superior ability to stabilize the emulsions, and remarkably, the emulsions stabilized by 2% proteins and 3:7 oil/water ratio efficiently resisted the freeze-thaw treatment and the change of pH (3–9), salt addition (NaCl, 50–400 mM), and storage temperatures (4–60°C). Furthermore, the emulsions showed a matched long-term stability with the existing biopolymers-stabilized emulsions. Consequently, this is the first finding of the by-product proteins in the head of giant freshwater prawn as an excellent emulsifier to stabilize emulsions, which help to improve the stability of food-grade emulsions and the added value of aquatic products.

## Introduction

Giant freshwater prawn (*Macrobrachium rosenbergii*) is one of the most significant freshwater species in the world. At present, its annual yield is about 250,000 tons (1). With the giant freshwater prawn farming in China, its aquaculture industry has been developing continuously because of its high commercial value. It is reported that the whole giant freshwater prawn contains 69–71% moisture, 25–26% protein, 1–2% lipid, and about 2% ash and carbohydrates (2).

Giant freshwater prawn is a good source of healthy food for human consumption and animal proteins for livestock (3). However, the head of giant freshwater prawn is often treated as a waste during the consumption and industrial processing. In fact, the head is also rich in proteins with high nutritional value and specific functional characteristics (4), but its utilization is rather low with only a small part being feed or fertilizer (5). Therefore, recycling the proteins in the head of giant freshwater prawn will facilitate the comprehensive utilization of aquatic resources.

Emulsions are stable systems formed by a certain proportion of oil phase, aqueous phase and emulsifiers. The emulsions have been widely used in food products, owing to their high stability, excellent antibacterial properties, and high nutritional value (6). The emulsifiers are used as surface-active substances and are dispersedly wrapped around the droplets. The emulsifiers contain both hydrophilic and oleophilic groups, which can adsorb at the water-oil interface to reduce the interfacial tension and hence maintain the stability of emulsions. Many chemicals are used as emulsifiers to prepare emulsions (7), but it is inappropriate to use them in the food industry because of their potential safety risks. Therefore, natural food grade emulsifiers show great advantages in stabilizing emulsions, promoting oil-aqueous phase dissolution, improving nutritional value and safety (8). Many studies prove that plant protein particles, such as corn protein (9), soybean protein (10), and pea protein (11), can be used as natural emulsifiers in food industry. However, plant proteins often lack key essential amino acids, and their high hydrophobicity makes them less likely to bind to water or oil (12). Generally, animal proteins generally contain eight essential amino acids, and their nutritional value is higher than plant proteins (13). Therefore, developing the proteins in the head of giant freshwater prawn as a food-grade emulsifier conforms to people's demand for “balanced diet,” and the concept of sustainable development.

On this basis, the proteins were extracted from the head of giant freshwater prawn by isoelectric precipitation, and their physicochemical properties were analyzed by amino acid analyzer, contact angle measurement, particle size measurement and size exclusion high performance liquid chromatography. Moreover, the functional ability of the proteins to stabilize emulsions was also explored under the conditions of freeze-thaw treatment, pH (3–9), salt addition (NaCl, 50–400 mM), and storage temperature (4–60°C).

## Materials and Methods

### Materials

Giant freshwater prawns were purchased from the Shanghai Aquaculture Base (Shanghai, China), and sunflower seed oil (Mighty, Suzhou, China) was purchased from a local supermarket (Mighty, Shanghai, China). All other materials and reagents were purchased from commercial suppliers and were used as received without any further purification.

### Extraction and Analysis of the Head Proteins of Giant Freshwater Prawn

#### Preparation of Protein Samples

The proteins were extracted from the head of giant freshwater prawn by isoelectric precipitation (14). The head of giant freshwater prawn were homogenized by homogenizer (SUOTN, STSRH-200, 800 rpm) with deionized water at the solid-to-solvent ratio of 1:5 (w/v). pH value of the homogenate was adjusted to 12 with 1 M NaOH and then stirred for 2 h. After 15 min centrifugation at 7,500 × g (RCF), the supernatant was adjusted to pH 4 with 1 M HCl, to yield precipitated proteins. The above operation was repeated three times to remove impurities, and then the aqueous protein dispersions were adjusted to pH 7 before freeze-drying. The protein content was measured by Kjeldahl method, and 6.25 was used as Kjeldahl nitrogen-to-protein conversion factor.

#### Amino Acid Analysis

After the hydrolysis of samples with 6 M HCl for 24 h, amino acid composition was analyzed by an HPLC system with pico-tag column. Hitachi D-2850 chromatograph data processor was used to quantify the amino acids according to the peak area of known standard concentration (15).

#### Size Exclusion High Performance Liquid Chromatography

SE-HPLC was carried out on a HPLC system (Agilent Technologies, United States) with a TSKgel G3000SWxL column (TOSOH Bioscience, Japan). The protein solution (pH 12.0, 2 mg/ml) was filtered by using a 0.45 μm membrane before injection. A mobile phase consisting of 0.1 M NaCl (pH 12.0) was used to elute the sample at an equidistant flow rate of 0.5 ml/min, and the absorbance was determined at 280 nm. SDS (15 mM) and DTT (25 mM) were added to the protein samples to analyze the protein aggregation (16).

#### Contact Angle Measurement

OCA 20 AMP (Dataphysics Instruments GmbH, Germany) was used to measure the contact angle of the proteins. The freeze-dried protein powder was pressed into a circular sheet and placed in an optical glass colorimetric dish containing sunflower seed oil. Then, 5 μl distilled water was dropped onto the sample surface with a high-precision syringe. After standing for 4 min, the image of the droplet was captured by a high-speed camera, and the contact angle was calculated by Laplace-Young equation (17).

#### Particle Size Distribution

The particle size distribution of proteins in aqueous dispersions was determined by dynamic light scattering using a Nanosizer SZ90 analyzer (Malvern Instruments, United Kingdom). A sample solution of 0.3 mg/ml (1.1 ml, pH 7.0) prepared in distilled water was used for measurement in a quartz colorimeter with an optical path of 1 cm (18). The refractive indices of water and the particles were taken as 1.33 and 1.54, respectively.

### Preparation of Emulsions

Emulsions were prepared using different protein solutions (1.0, 2.0, and 3.0%, wt) and different oil/aqueous phase ratios (2:8, 3:7, 4:6, and 5:5). Briefly, sunflower seed oil was added to protein solutions, homogenized at 16,000 rpm for 2 min using a rotor high speed blender (SUOTN, STSRH-200, China), and further treated by ultrasound processor (NingBo Scientz Biotechnology Co. Ltd., Ningbo, China) for 5 min (on 5 s, off 5 s, 300 W). The emulsions were prepared at pH 7.0 in the presence of sodium azide (0.02%, wt) to prevent the microbial contamination (19).

The effects of pH, salt addition and temperature on the stability of emulsions were investigated. The pH of protein solutions (2%, wt%) was adjusted to pH 3, 5, 7, and 9 with 1 M HCl or 1 M NaOH, and NaCl was added to the protein solutions (pH 7.0) to reach 50, 100, 200, and 400 mM, respectively. According to the above method, the emulsions were prepared with 2% protein and 3:7 oil/water ratio. At the same time, the emulsions (pH 7.0) were stored at different temperatures (4, 25, 37, 50, and 60°C) to investigate their stability.

### Creaming Index

The freshly prepared emulsions were stored at −20°C for 20 h, and then thawed in 20°C water bath for 2 h. After several freeze-thaw cycles, the height of emulsions and creaming layer was measured. Creaming index (CI) was calculated to reflect the freeze-thaw stability of emulsions by following equation (20).

$$\begin{array}{l} CI=\frac{H_{s}}{H_{t}}\times 100\% \end{array}$$

where, *H*_*s*_ is the height of the cream layer (cm); *H*_*t*_ is the total height of the emulsion (cm).

### Optical Microscope and Confocal Laser Scanning Microscope (CLSM)

The emulsions were diluted and dropped onto the microscope slide, and then directly observed under an optical microscope using a 63 × oil immersion (MC02709, Carl Zelss, Germany).

The emulsions were dyed with fluorescent dyes of Nile Blue (0.1 wt%) and Nile Red (0.1 wt%, W/V). The samples were placed on a recessed sheet under dark conditions, and then observed with CLSM (TCS SP8, Leica, Germany) using Helium Neon laser (633 nm) and Argon Krypton laser (488 nm) (21).

### Droplet Size Distribution and ζ-Potential of Emulsions

The droplet size distribution was determined by static light scattering using a Malvern Mastersizer 3000 analyzer (Malvern Instruments, United Kingdom). The samples were diluted with deionized water and stirred at a speed of 2,000 r/min to prevent multiple scattering effects. The refractive indices of water and sunflower seed oil were taken as 1.33 and 1.47, respectively. The Sauter, surface-weighted mean diameter (d_3,2_) was calculated as:

$$\begin{array}{l} d_{3,2}=\sum n_{i}d_{i}^{3}/\sum n_{i}d_{i}^{2} \end{array}$$

where, *n*_*i*_ is the number of droplets with diameter *d*_*i*_. Nano ZS90 Zetasizer was then used to measure the ζ-potential potential of the emulsions after 100 times dilution (22).

### Low-Field Nuclear Magnetic Resonance

The relaxation time of low field nuclear Magnetic resonance (LF-NMR) was measured using a MicroMR-22 MHz spectrometer (MicroMR, Shanghai, China). Five milliliters of the sample were placed into a 15 mm (diameter) NMR tube and the data was obtained from 6,000 echoes through 32 repeated scans. Carr-purcell-meiboom-gill sequence (CPMG) was used to determine the spin-spin relaxation time (23).

### Rheology of Emulsions

The rheological behavior of the emulsions was determined by a rheometer (AR 1500, TA Instruments, United Kingdom) in the frequency range of 1–10 rad/s. The parallel plate (40 mm diameter) gap of the measurement system was fixed to 1 mm, and the temperature was 25°C. The static emulsion was injected into the gap of the plate to conduct strain scanning, and the scanning frequency was tested at 0.1–10 Hz. The changes of storage modulus (G') and loss modulus (G”) were recorded (24).

### Data Analysis

The results were expressed as mean and standard deviation. SPSS software (IBM 20.0) was used to analyze the difference between the groups (*P* < 0.05).

## Results and Discussion

### Characterization of the Proteins in the Head of Giant Freshwater Prawn

#### Amino Acid Composition

The amino acid composition of protein determines the structure and function of protein. Therefore, it is of great significance to clarify the protein content and amino acid composition. In this study, the protein content in the head of giant shrimp was 69.1%, and the essential amino acid (EAA) content was 41.09% (Table 1). On this basis, the proteins greatly satisfied human growth and maintenance, compared with the EAA of soybean protein isolate (36.12%) (15), zein (39.34%), and gliadin (25.13%) (25).

**Table 1:** Amino acid composition of the proteins in the head of giant freshwater prawn.

**Amino acid**	**Percent amino acid (%)**
Asp	11.36
Thr	4.94
Ser	4.81
Glu	16.71
Pro	3.90
Ala	4.82
Gly	5.11
Cys	0.58
Val	4.81
Met	2.58
Ile	4.38
Leu	8.51
Tyr	4.68
Phe	5.02
Lys	7.32
His	3.53
Arg	6.66
Trp	0.28

#### Physicochemical Properties

**Figure 1:**
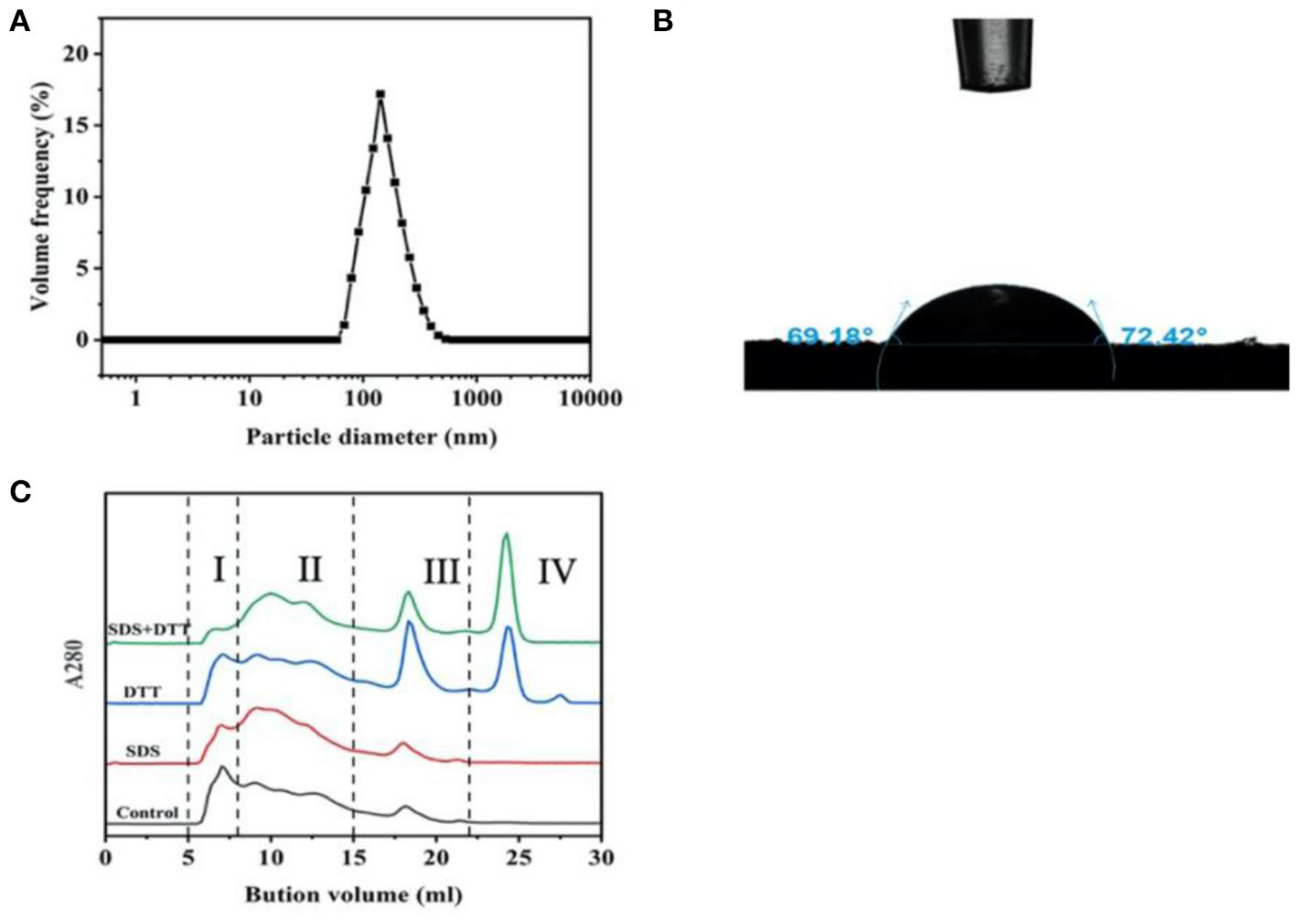
Particle size distribution **(A)**, contact angle **(B)**, and aggregation behavior **(C)** of the proteins in the head of giant freshwater prawn.

The particle size distribution of protein particles is shown in Figure 1A. It was monomodal, the particle size ranged from 100 to 500 nm and the *Z*-average was 141 nm. The particle size is very similar to those of Antarctic krill proteins (175 nm) (19), soy protein isolate-chitosan nanoparticles (148 nm) (18), novel zein particle (149 nm) (26) which possess a good ability to stabilize emulsions. Generally speaking, protein particles with smaller size present fast adsorption kinetics, which often quickly adsorb to the interface to form a more stable emulsion. In addition, the contact angle of protein particles was measured as ~71° which was <90° (Figure 1B), indicating that the protein particles were relatively hydrophilic and preferentially resided in the aqueous phase to form O/W emulsions (17).

#### Aggregation Behavior

In Figure 1C, the role of hydrophobic interaction and disulfide bond in proteins aggregation was determined after treatment with hydrophobic interaction denaturant SDS and disulfide bond reducing agent DTT (16). The elution profile consisted of four components, including high molecular weight protein (HMW, I), large molecular weight protein (LAMW, II), medium molecular weight protein (MMW, III) and low molecular weight protein (LMW, IV). In the control sample, the peak value of HMW (I) was at ~7 ml, and the peak value of LAMW (II) was at ~17 ml (MMW, III). When SDS was added, the peak intensity of HMW (I) decreased and peak intensity (LAMW, II) appeared at ~10ml. The peak intensity of MMW (III) had no obvious change. These results indicated that hydrophobic interactions contributed to the formation of protein aggregates, especially high molecular weight ones. After DTT treatment, the peak intensities of HMW (I) and LAMW (II) decreased slightly, while the peak intensities of MMW (III) and LMW (IV) increased sharply. In the presence of SDS and DTT, the peak of HMW (I) almost disappeared, and the corresponding LMW (IV) of monomer increased significantly, while the peak intensity of (LAMW, II) and MMW (III) decreased slightly. These results further indicated that hydrophobic interactions and disulfide bonds promoted the aggregation of HMW (I), and disulfide bonds were more important than hydrophobic interactions in maintaining the stability of protein conformation.

Hydrophobic interactions occurred through the interaction of hydrophobic amino acids (including Gly, Ala, Val, Leu, Ile, Phe, Trp, Met, Pro), which is the driving force of protein folding (27). In this study, hydrophobic amino acids accounted for 39.41% of the total amino acids. Under these conditions, the hydrophobic side chains of the proteins were embedded in the hydrophobic core, exposing the hydrophilic group to the environment (28). Therefore, it could be highly inferred that this protein conformation determined the wettability of the head protein of giant freshwater shrimp (Figure 1B). In addition, the content of cysteine was 0.58%, which greatly promoted the formation of disulfide bond. Our previous study found that Antarctic krill protein contained 40.81% hydrophobic amino acids and 0.28% cysteine residues. Hydrophobic interaction played a greater role in promoting protein aggregation than disulfide bond (10). This difference is mainly due to the fact that the cysteine content in the head of giant freshwater shrimp is more than twice that of Antarctic krill.

### Ability of the Proteins in the Head of Giant Freshwater Prawn to Stabilize Emulsions

**Figure 2:**
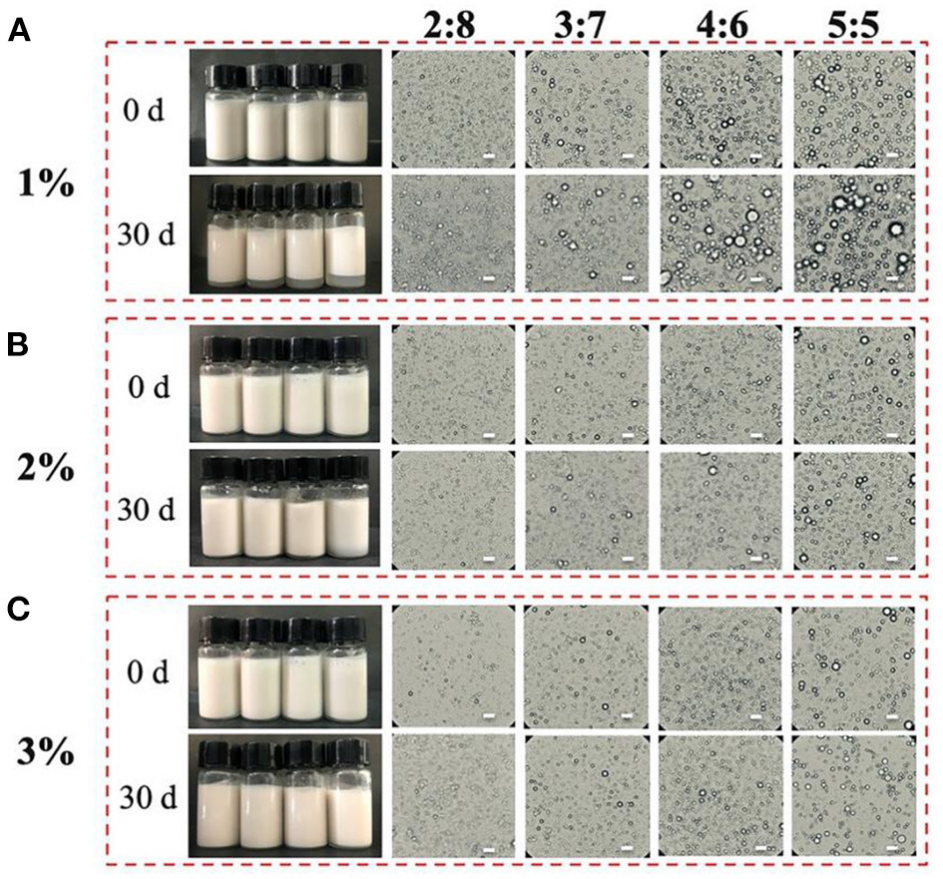
Visual appearance and microstructures of the proteins-stabilized emulsions with different concentrations **(A–C)** at initial (0 days) and after 30 days of quiescent storage. Scale bar = 5 μm.

The protein concentrations (1–3%, W/V) and oil/water ratios (2:8–5:5, V/V) were selected to prepare stable oil/water emulsions. Figure 2 shows the appearance and microstructures of the proteins-stabilized emulsions. In Figure 2A, the emulsions prepared by 1% proteins displayed creaming after 30 days storage. Furthermore, the emulsions creamed more obviously with the increase of oil/water radio from 2:8 to 5:5. After the proteins was increased to 2 or 3%, the emulsions visually unchanged and did not present creaming (Figures 2B,C). From the viewpoint of microstructures, the droplet diameter of 1% proteins-stabilized emulsions significantly increased with the increase of oil/water radio after 30 days storage. However, the 2 or 3% proteins-stabilized emulsions displayed similar microstructures with each other during the whole storage. The increased diameter indicated that the coalescence occurred in the emulsions. The reason was that under low concentration of protein particles, the increase of oil/water radio caused insufficient protein particles to absorb to the surface of the droplets, thereby increasing the surface area of oil droplets to maintain the stability (29). With the increase of protein content, higher particle density resided on the O/W interface to achieve sufficient surface coverage of all droplets (18). Comparing with plant proteins including tea protein (30), soy protein isolate (31), zein (26), and animal proteins including Antarctic krill proteins (19), whey proteins (32), pork proteins (33), the emulsions stabilized by the proteins in the head of giant freshwater prawn also show excellent and comparable stability after a long-term storage.

**Figure 3:**
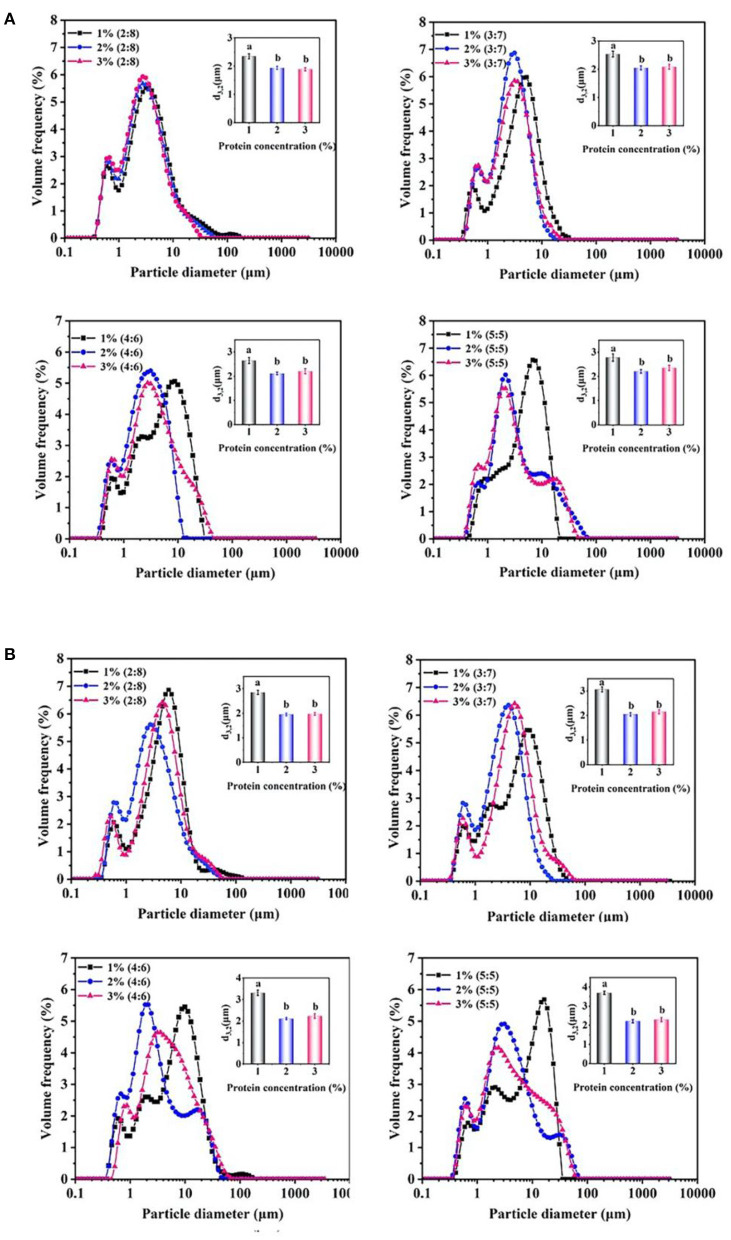
Droplet size changes of the proteins-stabilized emulsions after 0 days **(A)** and 30 days **(B)**. Mean values followed by different letters are significantly different (*P* < 0.05).

As shown in Figure 3A, the average diameter (d_3,2_, μm) of 1% proteins-stabilized emulsions was 2.6 μm at 2:8 oil/water ratio. When the oil/water ratio was 5:5, the droplet size increased to 2.8 μm. However, the emulsion prepared by 2 or 3% proteins remained ~2.0 μm in diameter with the increase of oil/water radio from 2:8 to 5:5. After 30 days storage (Figure 3B), the droplet sizes of 1% proteins-stabilized emulsions greatly increased reaching 2.8, 3.1, 3.3, and 3.7 μm at oil/water ratios of 2:8, 3:7, 4:6, and 5:5, respectively. However, 2 or 3% proteins-stabilized emulsions almost showed no change in droplet sizes (~2.0 μm) under different oil/water ratios. This further suggested that the emulsions prepared by 1% proteins underwent the coalescence during storage. Considering the stability of emulsions and the added amount of proteins, 2% proteins-stabilized emulsions were used to perform subsequent experiments.

**Figure 4:**
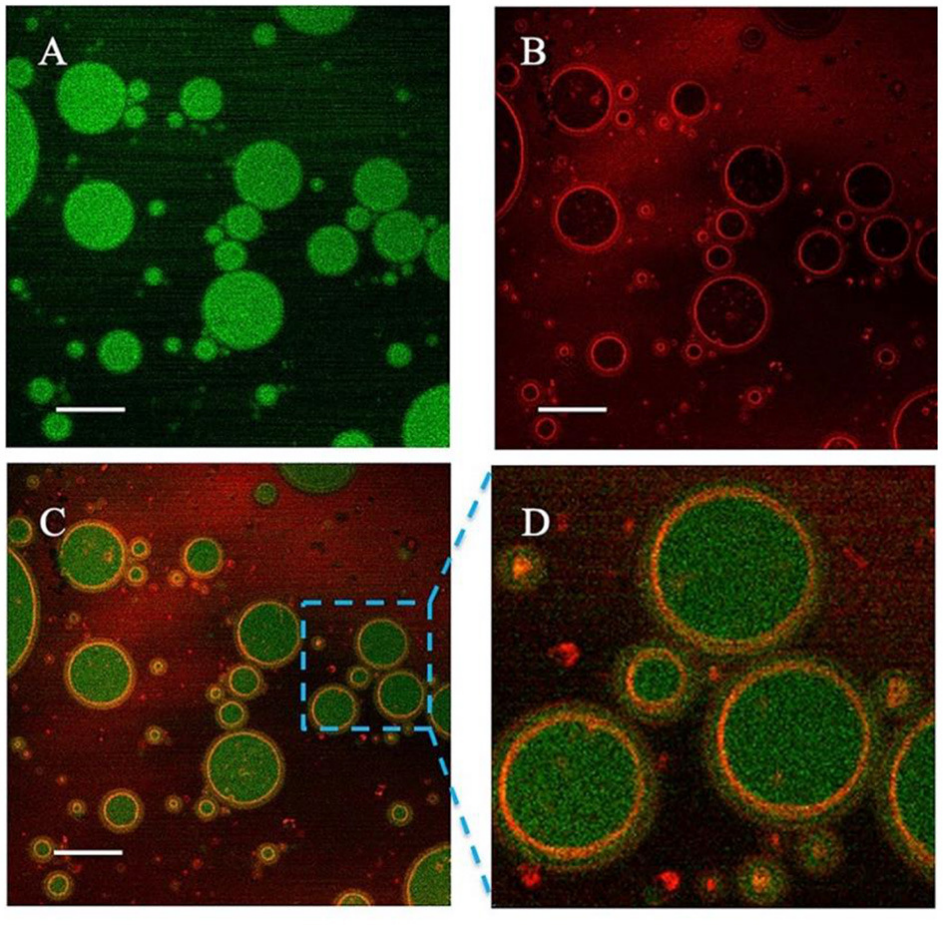
Confocal laser scanning microscope (CLSM) images of the proteins-stabilized emulsions. **(A)** Nile red fluorescence (green) labeling Sunflower seed oil excited; **(B)** Nile Blue fluorescence (red) labeling proteins; **(C)** The overlapped image; **(D)** Enlarged image. Scale bar = 5 μm.

The emulsion microstructure was characterized by CSLM. Figure 4A shows the green fluorescence images of sunflower seed oil stained with Nile Red, and Figure 4B shows the red fluorescence images of protein stained with Nile Blue. Therefore, the green and red fluorescence represent oil phase and proteins particles, respectively. Figures 4C,D are the overlapped images of Figures 4A,B. It was clearly observed that the red fluorescence was wrapped around by green fluorescence, which supported the O/W type of emulsion. Moreover, the proteins stained by red color were uniformly and tightly distributed around the oil droplets, which effectively prevented the coalescence (32).

### Effects of Different Environmental Stresses on the Stability of Emulsions

#### Effect of Freeze-Thaw Treatment

**Figure 5:**
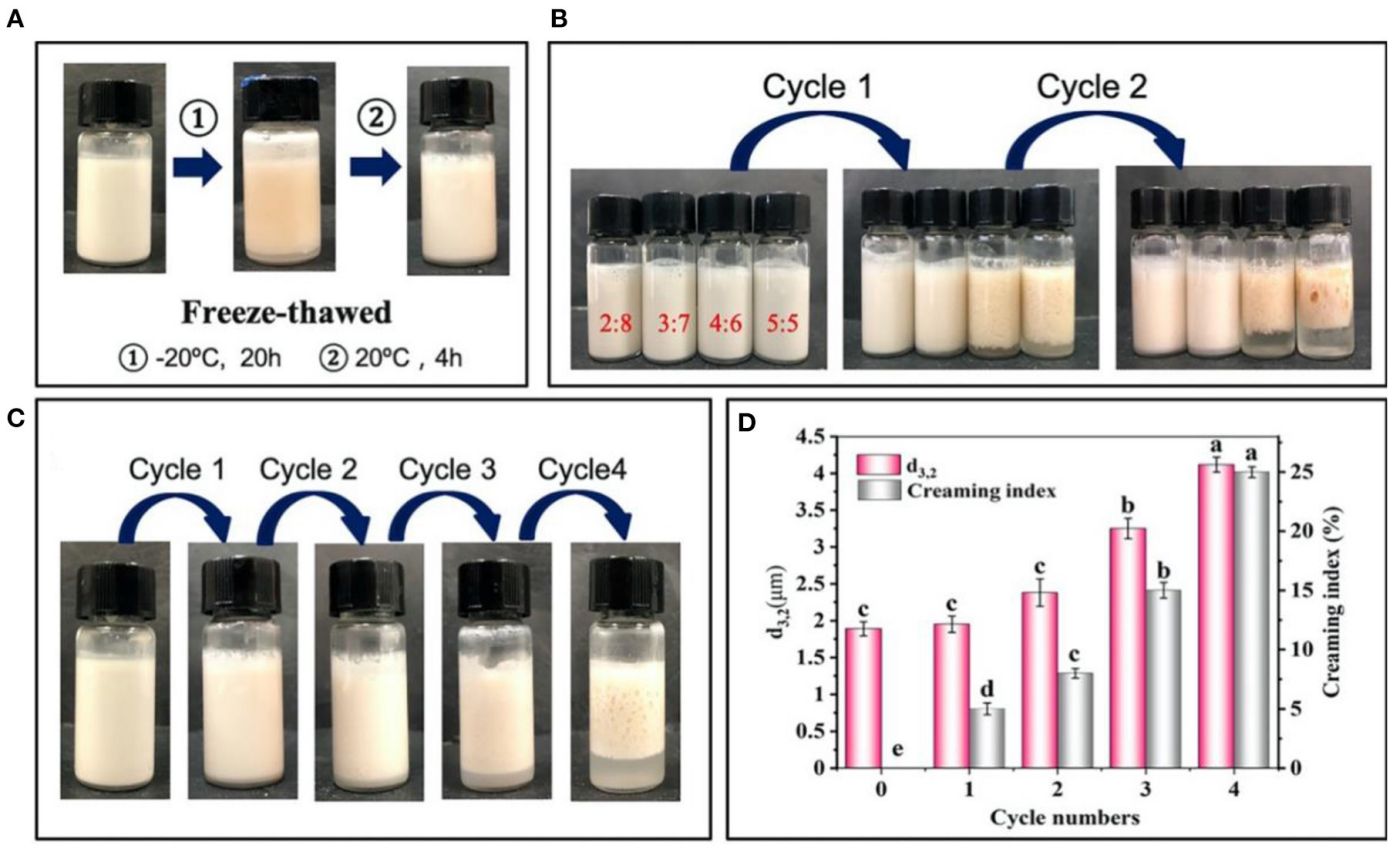
Changes in visual appearance **(A,B)** of emulsions prepared by 2% proteins and 3:7 oil/water ratios **(C)** before and after freeze-thaw treatment. **(D)** Average diameter (d_3,2_) and creaming index of emulsions prepared by 2% proteins and 3:7 oil/water ratio after freeze-thaw treatment. Mean values followed by different letters are significantly different (*P* < 0.05).

The emulsions prepared by 2% proteins were treated by the freeze-thaw treatment (Figure 5A), and their stability was further evaluated by the creaming index and d_3,2_ values of the droplets. Figure 5B shows the changes in the stability of emulsions after two cycles freeze-thaw treatment. In detail, the emulsions at the oil/water ratios of 4:6 and 5:5 were creamed after one cycle, and further displayed an obvious demulsification after two cycles, suggesting that the adsorbed protein particles greatly desorbed from the oil/water interface. With the emulsions being frozen, the water molecules formed ice crystals puncturing interfacial membrane, which induced the oil phase to penetrate each other and then gathered leading to the separation of oil phase and aqueous phase after thawing (34). On this basis, the emulsions at higher oil/water ratio (e.g., 4:6 and 5:5) formed larger droplets after being frozen, which was difficult to be completely covered by the limited proteins (35). Therefore, the emulsions at lower oil/water ratios of 2:8 and 3:7 exhibited higher freeze-thaw stability (Figure 5B). After further freeze-thaw treatment of the emulsions at 3:7 oil/water ratio (Figure 5C), obvious creaming was observed after 3 cycles and the creaming index maximally reached 25% after four cycles treatment. Moreover, a high degree of demulsification also occurred. Meanwhile, the d_3,2_ values of the emulsions increased from initial 1.9–4.1 μm (Figure 5D). The results showed that the appropriate oil/water ratio and proteins concentration was 3:7 and 2%, which was selected to prepare the emulsions with a long-term stability and high freeze-thaw resistance.

#### Effect of pH

**Figure 6:**
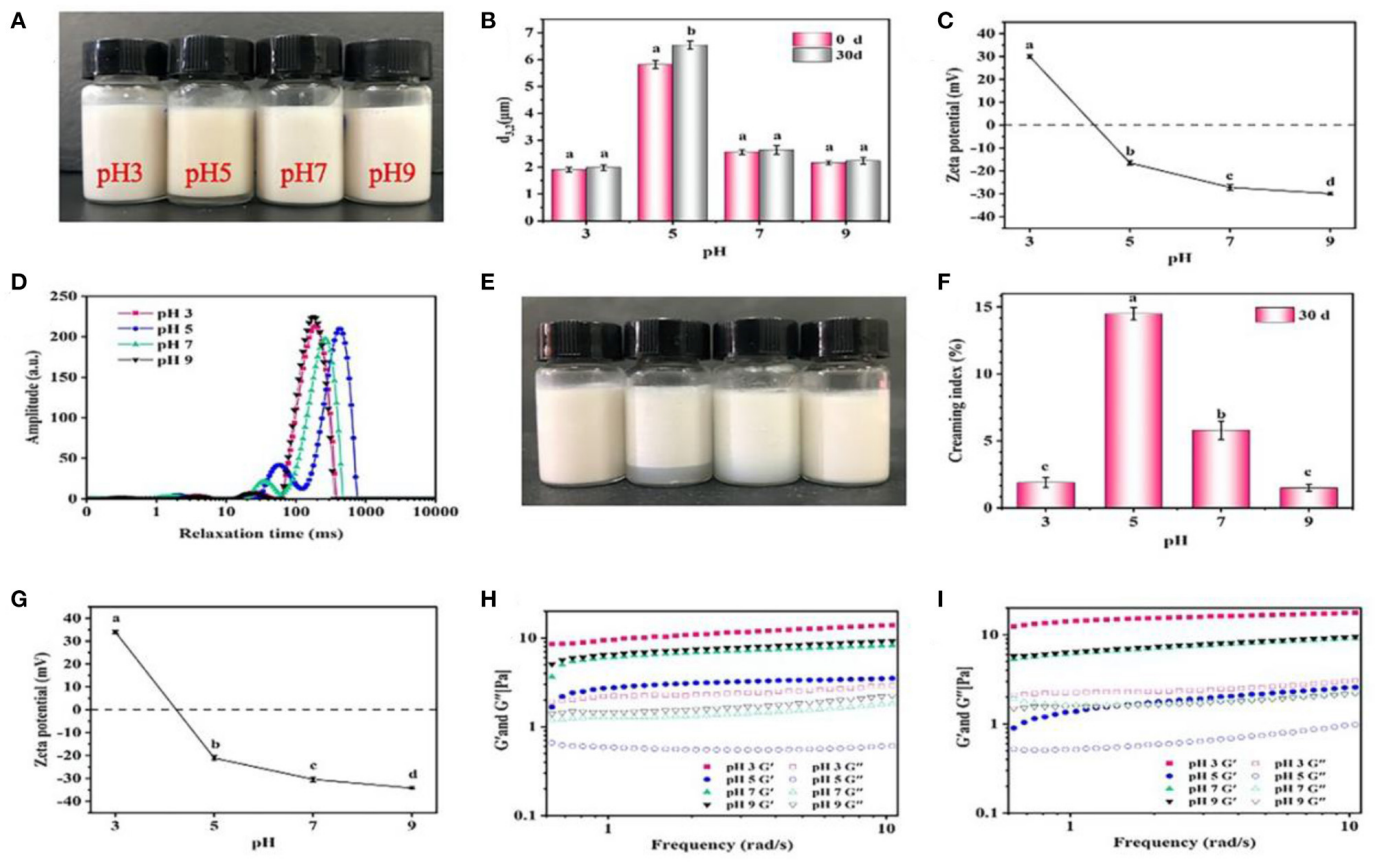
Effects of pH on the visual appearance **(A,E)**, droplet size **(B)**, ζ-potential **(C,G)**, LF-NMR **(D)**, creaming index **(F)**, and rheological properties **(H,I)** of the emulsions prepared by 2% proteins and 3:7 oil/water ratio after 0 and 30 days storage. Mean values followed by different letters are significantly different (*P* < 0.05).

In general, the visual appearance of the freshly prepared emulsions was similar with each other at different pH values, and only a slight creaming occurred when pH was 5 (Figure 6A). The emulsions showed smaller d_3,2_ values (~2.0 μm) at pH 3, 7 and 9 compared with those (5.8 μm) at pH 5 (Figure 6B). Furthermore, the ζ-potential values at different pH were analyzed (Figure 6C). When the pH value was 3, 7, and 9, the ζ-potential values were >30 mV, and when the pH value was 5, a minimum 20.2 mV was reached. Previous studies have shown that pH 5 is close to the isoelectric point of giant shrimp protein, which reduces the surface charge of protein and forms aggregates (16). However, the proteins had a high positive charge at pH 3, and a high negative charge at pH 7 and 9, thus inhibiting the aggregation of proteins and maintaining the stability of emulsions. The relaxation time was rather sensitive to the mobility of water molecules, which was an important index reflecting the water migration during the formation of emulsions (Figure 6D). All of the emulsions showed two main relaxation peaks, which belonged to constitution water (10–100 ms) and movable water (100–1,000 ms), respectively. In contrast, the relaxation peaks shifted to the right at pH 5, suggesting that the mobility of the emulsions increased and its stability decreased (23). After 30 days storage, the visual appearance of emulsions did not change significantly at pH 3, 7, and 9 (Figures 6E,F), and the droplets size reached the minimum value of 1.9 μm at pH 3 and the maximum value of 6.5 μm at pH 5. Meanwhile, the ζ-potential of emulsions did not greatly change (Figure 6G). Such phenomenon was mainly because that when the pH reached the pI, the electrostatic repulsion between droplets decreased, which induced the aggregation of droplets and creaming (36).

The rheological properties of emulsions are shown in Figure 6H. Under different pH conditions, the storage modulus G' of all emulsions was higher than the loss modulus G”, and all showed an increasing trend with the increase of shear frequency, which manifested that the gel-like networks were weak or similar to solid structure (20). Moreover, the G' and G” were lower at pH 5 than those at pH 3, 7, and 9. Generally, the higher the G' and G” values, the more stable the gel-like networks will be. These results were consistent with the LF-NMR results, because the increased mobility of emulsions weakened the gel-like networks (37). After 30 days storage, the G' and G” at pH 3, 7, and 9 further rose (Figure 6I). Such facts were mainly because that when the pH was far away from the pI, the proteins became charged and electrostatic repulsion increased, thereby forming small and flexible aggregates to stabilize emulsions with low d_3,2_ values (19). Meanwhile, the volume of pores between emulsion droplets was decreased and the O/W interface contact area was increased, which induced the formation of more compact network structures and the increase of viscoelastic properties (24). In addition, the formation of gel-like networks was also contributed by the intermolecular interactions between proteins. For example, the hydrophobic interactions facilitated the adsorption and aggregation of protein particles at the O/W interface, and disulfide bonds could further stabilize protein molecules at the O/W interface to enhance their interactions, which facilitated the formation of the stabilized emulsions (38). Considering the results from SE-HPLC, it was inferred that hydrophobic interactions improved the formation of emulsions, and the disulfide bonds greatly maintained their stability.

#### Effect of Salt Addition and Temperature

**Figure 7:**
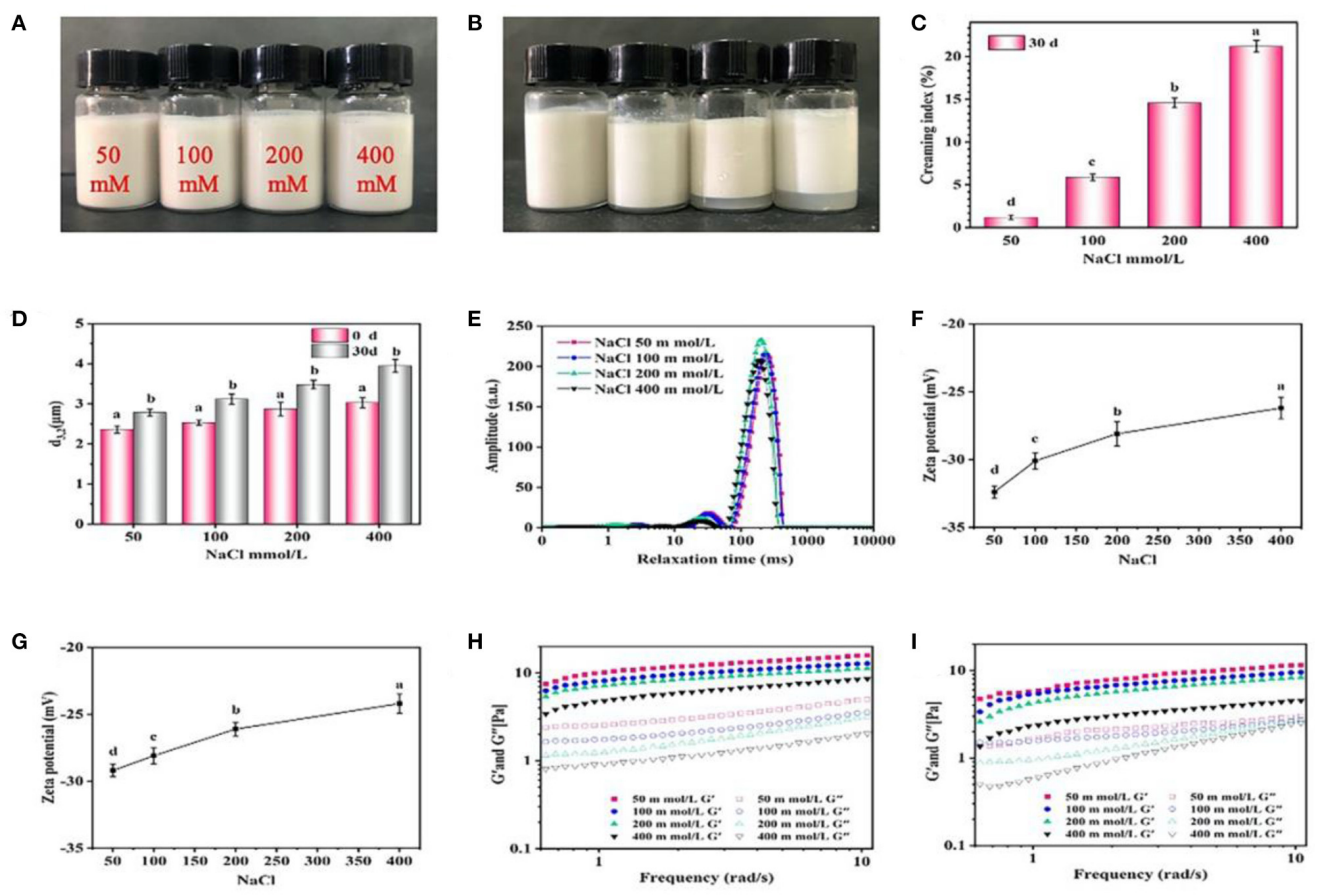
Effects of NaCl concentration on the visual appearance **(A,B)**, creaming index **(C)**, droplet size **(D)**, LF-NMR **(E)**, ζ-potential **(F,G)** and rheological properties **(H,I)** of the emulsions prepared by 2% proteins and 3:7 oil/water ratio after 0 and 30 days storage. Mean values followed by different letters are significantly different (*P* < 0.05).

The effect of salt addition on stability of emulsions is displayed in Figure 7. No obvious creaming occurred in the freshly prepared emulsions at different NaCl (50–400 mM) (Figure 7A), and the d_3,2_ values of emulsions increased from 2.4 to 3.0 μm (Figure 7B). The emulsions with a higher concentration of NaCl (>200 mM) was more obviously creamed (Figure 7C), and the d_3,2_ values also increased to 3.9 μm after 30 days storage (Figure 7D). Figure 7E shows the distribution of relaxation time of the emulsions with different NaCl. The relaxation time shifted to the left with the increase of NaCl, indicating that the addition of NaCl restrained the mobility of emulsion droplets. There was a negative correlation between NaCl concentration and relaxation time of the emulsions, possibly due to that the salt provided a electrostatic shielding of the charged protein particles to promote proteins aggregation and limit the mobility of emulsions (39). This argument was supported by the decrease of ζ-potential with increasing the NaCl concentration, indicating that the electrostatic repulsion between droplets was weakened (Figures 7F,G). Furthermore, the values of G' and G” were greatly reduced with the increase of NaCl, and such phenomenon become more obvious compared with freshly prepared emulsions after 30 days storage (Figures 7H,I), suggesting that the electrostatic shielding of the charged particles weakened the formation of gel-like networks which led to the unstable emulsion.

**Figure 8:**
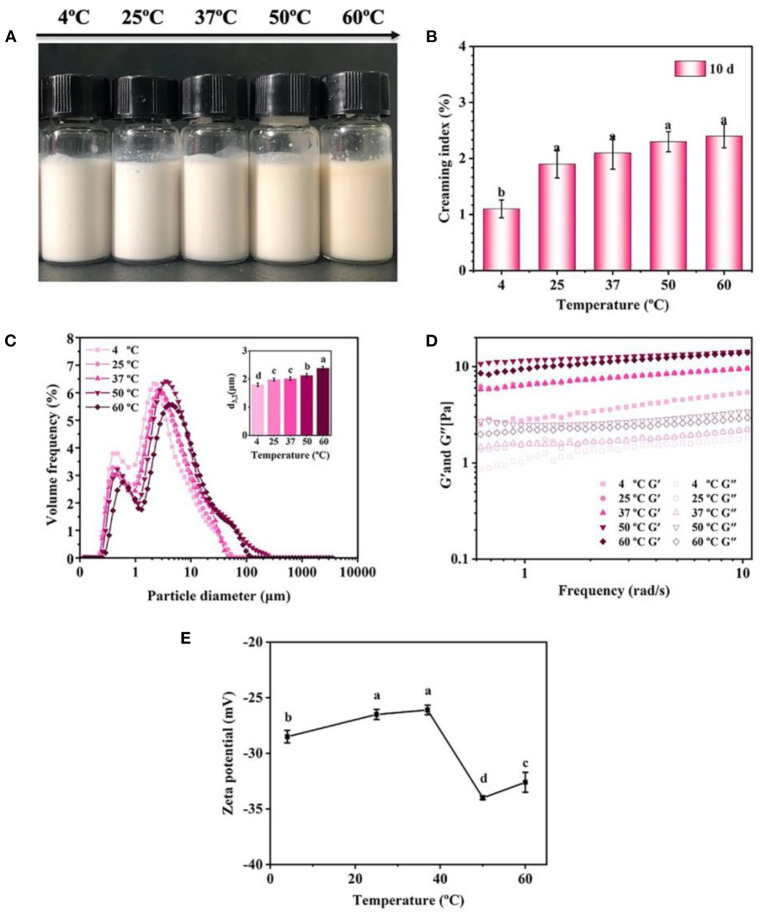
Effects of storage temperature on the visual appearance **(A)**, creaming index **(B)**, droplet size **(C)**, rheological properties **(D)**, and ζ-potential **(E)** of the emulsions (pH 7) prepared by 2% proteins and 3:7 oil/water ratio after 10 days storage. Mean values followed by different letters are significantly different (*P* < 0.05).

The effect of temperature on the stability of emulsions was evaluated (Figure 8). There was no obvious creaming or phase separation in the emulsions after 10 days storage at different temperature (4–60°C) (Figures 8A,B). The droplet size of emulsions slightly increased from 1.8 μm at 4°C to 2.4 μm at 60°C (Figure 8C). In addition, the increase of temperature had a great influence on the rheological properties of emulsion. In detail, the emulsions presented higher G' and G” values at 50 and 60°C compared with those at 4, 25, and 37°C (Figure 8D), indicating that the formation of gel-like networks was enhanced at 50 and 60°C. Generally, heating induced the unfolding of the proteins, causing the exposure of the side chain including hydrophobic groups, charged groups and cysteine residues etc., and then the exposed groups would either re-fold or re-form covalent/non-covalent intermolecular interactions with other molecules (27). Such deduction was also highly supported by the results of ζ-potential, showing that the emulsions treated by 50 and 60°C has a higher ζ-potential than those treated by lower temperatures (Figure 8E). Therefore, it was concluded that the heating improved the intermolecular interactions between the protein particles, which finally enhanced the formation of gel-like networks in the emulsions.

Wu et al. (40) found that when the pH was 5, the whey protein isolate-stabilized emulsions greatly aggregated and a phase separation was evidenced. Hu et al. (41) reported that the emulsions stabilized by rice bran proteins became unstable under 0–300 mM NaCl treatment, and creaming was easy to occur. Ding et al. (42) discovered that fish oil-loaded crosslinked gelatin nanoparticle emulsions were sensitive to temperature changes, and obvious phase separation was observed after 37°C. Therefore, the proteins in the head of giant freshwater prawn would be a good food-grade emulsifier to stabilize emulsion in the food industry.

## Conclusion

There were a lot of balanced EAA in the proteins in the head of giant freshwater prawn, with small particle size, intermediate wettability, and preferential absorption on the interface to form stable emulsions. On this basis, a novel protein-stabilized emulsion was developed, and notably, the emulsions stabilized by 2% proteins and 3:7 oil/water ratio effectively resisted the environmental stresses (freeze-thaw treatment, pH, salt addition, storage temperature). Thereinto, the hydrophobic interactions improved the formation of the proteins-based emulsions, and the disulfide bonds greatly maintained the stability of emulsions. In comparison with the existing biopolymers-stabilized emulsions, the proteins-based emulsions showed an excellent long-term stability. Therefore, this study not only proves the ability of the proteins in the head of giant freshwater prawn in stabilizing the emulsions, but also provides a new idea to develop the by-product proteins of aquatic products.

## Data Availability Statement

The original contributions presented in the study are included in the article/supplementary material, further inquiries can be directed to the corresponding author/s.

## Author Contributions

YW: investigation, software, resources, data curation, and writing—original draft. YL and RW: investigation, software, resources, and data curation. JW and YZ: conceptualization, funding acquisition, and writing—review and editing. HL: software, resources, and supervision. All authors contributed to the article and approved the submitted version.

## Conflict of Interest

The authors declare that the research was conducted in the absence of any commercial or financial relationships that could be construed as a potential conflict of interest.
